# Supplementary material for: Transcriptome Analysis Reveals Inhibitory Effects of Lentogenic Newcastle Disease Virus on Cell Survival and Immune Function in Spleen of Commercial Layer Chicks
Source: Genes (Basel). 2020 Aug 26;11(9):1003. doi: 10.3390/genes11091003 (PMC7565929; doi:10.3390/genes11091003)
Supplement: Supplementary file 1 [file genes-11-01003-s001.zip › Supplementary tables/Table S1.docx]

**Table S1. Statistical summary of sequence reading, mapping and counting**

| **Group** | **dpi** | **ID** | **Raw Reads** | **Mapped Reads** | **% of Mapped Reads** | **Detected Genes** | **Transcriptome Coverage**  **Coverage** |
| --- | --- | --- | --- | --- | --- | --- | --- |
| Control | 2 | 3554 | 13,236,228 | 12,094,372 | 91.4% | 17,287 | 70.98% |
| Control | 2 | 3851 | 12,094,372 | 11,514,742 | 89.8% | 17,263 | 70.88% |
| Control | 2 | 4453 | 15,036,732 | 13,497,921 | 89.8% | 17,447 | 71.63% |
| Control | 2 | 4591 | 13,519,405 | 12,096,793 | 89.5% | 17,301 | 71.03% |
| NDV | 2 | 3713 | 11,418,370 | 10,202,378 | 89.4% | 16,932 | 69.52% |
| NDV | 2 | 3770 | 12,509,809 | 11,183,751 | 89.4% | 17,078 | 70.12% |
| NDV | 2 | 3905 | 10,383,866 | 9,328,372 | 89.8% | 16,897 | 69.38% |
| NDV | 2 | 4522 | 13,337,707 | 11,967,529 | 89.7% | 17,306 | 71.05% |
| Control | 6 | 383111 | 15,892,353 | 14,357,032 | 90.3% | 17,389 | 71.40% |
| Control | 6 | 4060 | 13,946,096 | 12,467,437 | 89.4% | 17,463 | 71.70% |
| Control | 6 | 4352 | 15,409,270 | 13,898,239 | 90.2% | 17,155 | 70.43% |
| Control | 6 | 45511 | 16,783,037 | 15,073,251 | 89.8% | 17,317 | 71.10% |
| NDV | 6 | 42410 | 15,376,512 | 13,606,955 | 88.5% | 17,390 | 71.40% |
| NDV | 6 | 43510 | 12,719,693 | 11,463,903 | 90.1% | 17,010 | 69.84% |
| NDV | 6 | 44410 | 15,845,693 | 14,260,570 | 90.0% | 17,286 | 70.97% |
| NDV | 6 | 4490 | 14,706,371 | 13,071,212 | 88.9% | 17,551 | 72.06% |

**Note:** The percentage of mapped reads was calculated as the number of mapped reads divided by the number of raw reads; Transcriptome coverage was calculated as the number of detected genes (genes with at least one mapped read) divided by the total number of genes in Galgal6.0 genome which is 24356.
